# Planting Patterns and Deficit Irrigation Strategies to Improve Wheat Production and Water Use Efficiency under Simulated Rainfall Conditions

**DOI:** 10.3389/fpls.2017.01408

**Published:** 2017-08-22

**Authors:** Shahzad Ali, Yueyue Xu, Xiangcheng Ma, Irshad Ahmad, Muhammad Kamran, Zhaoyun Dong, Tie Cai, Qianmin Jia, Xiaolong Ren, Peng Zhang, Zhikuan Jia

**Affiliations:** ^1^Institute of Water Saving Agriculture in Arid Areas of China, Northwest A&F University Yangling, China; ^2^Key Laboratory of Crop Physi-ecology and Tillage Science in North-western Loess Plateau, Ministry of Agriculture, Northwest A&F University Yangling, China

**Keywords:** deficit irrigation, simulated rainfall, winter wheat yields, planting patterns, grain filling rate, WUE, economic profits

## Abstract

The ridge furrow (RF) rainwater harvesting system is an efficient way to enhance rainwater accessibility for crops and increase winter wheat productivity in semi-arid regions. However, the RF system has not been promoted widely in the semi-arid regions, which primarily exist in remote hilly areas. To exploit its efficiency on a large-scale, the RF system needs to be tested at different amounts of simulated precipitation combined with deficit irrigation. Therefore, in during the 2015–16 and 2016–17 winter wheat growing seasons, we examined the effects of two planting patterns: (1) the RF system and (2) traditional flat planting (TF) with three deficit irrigation levels (150, 75, 0 mm) under three simulated rainfall intensity (1: 275, 2: 200, 3: 125 mm), and determined soil water storage profile, evapotranspiration rate, grain filling rate, biomass, grain yield, and net economic return. Over the two study years, the RF treatment with 200 mm simulated rainfall and 150 mm deficit irrigation (RF2_150_) significantly (*P* < 0.05) increased soil water storage in the depth of (200 cm); reduced ET at the field scale by 33%; increased total dry matter accumulation per plant; increased the grain-filling rate; and improved biomass (11%) and grain (19%) yields. The RF2_150_ treatment thus achieved a higher WUE (76%) and RIWP (21%) compared to TF. Grain-filling rates, grain weight of superior and inferior grains, and net economic profit of winter wheat responded positively to simulated rainfall and deficit irrigation under both planting patterns. The 200 mm simulated rainfall amount was more economical than other precipitation amounts, and led to slight increases in soil water storage, total dry matter per plant, and grain yield; there were no significant differences when the simulated rainfall was increased beyond 200 mm. The highest (12,593 Yuan ha^−1^) net income profit was attained using the RF system at 200 mm rainfall and 150 mm deficit irrigation, which also led to significantly higher grain yield, WUE, and RIWP than all other treatments. Thus, we recommend the RF2_150_ treatment for higher productivity, income profit, and improve WUE in the dry-land farming system of China.

## Introduction

The semi-arid regions of northwest China are crucial for winter wheat production. In northwest China precipitation serves as the main water source, and most crop production depends on natural rainwater (Ren et al., 2016). However, the rainfall is often inadequate in these areas and average annual rainfall was 379 mm over a 48-year period (1966–2014) (Li and Gong, 2002). In addition, the raining season in this area does not overlap with the growth stages of wheat because 72% of rainfall occurs between July and September, and winter wheat is grown between October and June (Wen et al., 2012). This indicates a severe ecological problem for improving sustainable, dry-land farming. In addition, the annual evaporation is more than 800 mm, which leads to severe water deficiencies (Kang et al., 2003; Ren et al., 2016). To deal with the water scarcity issue, it is essential to implement water saving farming practices to optimize consumption of limited rainfall. Such practices include micro rainwater harvesting and water storage, both of which can enhance the WUE of winter wheat (Ren et al., 2008).

In semi-arid areas of China, it is hard to enhance plant growth due to water scarcity (Nagaz et al., 2012). Recently, the ridge furrow (RF) micro-rainfall collecting system has been extensively established in semi-arid regions. The RF planting model includes a rainfall collecting zone (ridge) and sowing zone (furrow) which improve precipitation use efficiency (Abbas et al., 2005). In addition, the RF system can decrease evaporation and provide adequate water at key growth stages of winter wheat by increasing soil moisture contents and increasing crop water use efficiency (Li X. Y. et al., 2001). Ren et al. (2016) also reported that the best simulated rainwater amount for the RF planting model is 230–440 mm, but the biomass accumulation, WUE and grain yield of maize did not increase significantly when precipitation was higher than 440 mm.

Water deficit during flowering and grain-filling stage causes considerable yield reductions in wheat which are primarily due to accelerated leaf senescence, oxidative damage to photo-assimilatory machinery, assimilate translocation, reduced grain set, and sink capacity (Farooq et al., 2014). Several researchers recommend that water scarcity during the critical growth stages (flowering and grain filling stage) of wheat can be reduced by the RF system as a result improve total dry matter and grain yield in semi-arid regions (Patrick et al., 2004). But, during some critical crop growth stages, water shortages are unavoidable (Pan et al., 2003). Deficit irrigation (DI) with micro-water collection through the RF system could be a practical solution to supply water during critical crop growth stages, and as a result, significantly increase dry matter accumulation, grain yield and WUE (Xiao et al., 2005). DI is a highly efficient practice with huge potential for improving crop productivity (Olesen et al., 2000; Fereres and Soriano, 2006). Recent studies have revealed that an optimized DI regime increased soil moisture critical crop growth stages as a result improve winter wheat yields (Guo et al., 2014). However, an excessive amount of irrigation leads to a high rate of evaporation (ET) and high dry matter per plant, but it does not produce the maximum seed yield and reduces the WUE and irrigation water productivity (Kang et al., 2002; Geerts et al., 2008). Ren et al. (2008) showed that with various simulated rainfall conditions, i.e., 230, 340, and 440 mm, the spring maize yield was increased with ridge and furrow rainfall harvesting cultivation by 82.8, 43.4, and 11.2%, respectively, while the water use efficiency was increased by 77.4, 43.1, and 9.5%, when compared with flat cultivation without ridge and plastic.

The RF system combined with plastic film mulching can increase the soil water availability for crops by collecting water from low intensity rainfall events, and preserve surface runoff during heavy precipitation thereby facilitating sustainable farming productivity and high water use efficiency (WUE) in semiarid areas of the world (Jia et al., 2006). The RF system also reduces soil evaporation and enhances rainwater penetration (Li F. M. et al., 2001; Xiao et al., 2005). Moreover, Wang et al. (2015) demonstrated that RF system is practical and significantly increases the consumption of rainfall, WUE and biomass for wheat. The RF system combined with limited irrigation has significantly enhanced crop yield in semi-arid regions (Xiao and Wang, 2003). However, the RF system has not been promoted widely in semi-arid regions, which are primarily remote, hilly areas (Hu et al., 2002). To exploit its efficiency on large-scale, the RF system needs to be tested with different amount of simulated precipitation with deficit irrigation. Therefore, to maximize the consumption of rainfall during light precipitation events in semi-arid regions, we tested two planting patterns with three deficit irrigation levels under simulated rainfall conditions. This test allowed us to investigate the effect of the RF planting model with deficit irrigation on yields and rainfall irrigation water productivity (RIWP).

To attain higher winter wheat yields, farmers in semi-arid areas pump groundwater to balance the ET deficit. More than 80% of the groundwater resources in China have been used for irrigation (Huang et al., 2005). The unnecessary use of groundwater resources in the semi-arid areas causes the water table to drop and produces many other ecological issues (Ali and Thei, 2004). The groundwater table is dropping gradually at the rate of 1 m year^−1^ in semi-arid of China, and the major cause of this reduction is the increasing winter wheat growing area. Winter wheat is irrigated with groundwater, but has a low WUE (Hu et al., 2002). Thus, stopping it is essential to stop the water table from dropping by reducing the amount of groundwater through the use of different planting patterns under simulated precipitation with deficit irrigation in dry-land farming systems.

The potential yield of winter wheat can be determined by three major components: grain weight, grains spike^−1^, and spikes plant^−1^. However, improving the grain-filling rate is more important in semi-arid regions (Yang et al., 2006). The inadequate supply of deficit irrigation and unnecessary losses of insufficient precipitation leads to decreased winter wheat yields in dry-land farming systems. The RF system combined with deficit irrigation under simulated rainfall conditions is gradually becoming more common in semi-arid areas (Ogola et al., 2002). The purpose of this study is: (1) to assess the effect of RF system with deficit irrigation levels on winter wheat grain yield, grain-filling and WUE under simulated rainfall conditions (2) to explore the influence of RF system with deficit irrigation levels on soil water storage, ET, RIWP, and economic returns under simulated rainfall conditions. The results obtained are in the hope to produce scientific support for giving useful guidelines to farmers of semi-arid regions on how to optimize agro-management practices for water-saving and high-yielding winter wheat cultivation.

## Materials and methods

### Site description

This research work was carried out from 2015 to 2017 at Northwest A&F University, Yangling, Shaanxi Province, China. The research site was situated at the longitude of 108°24′E and latitude of 34°20′N, and was 466.7 m above sea level. The climatic conditions at the study location are semi-arid with a warm temperate and an annual average evaporation rate of 1,753 mm. Annual mean temperature is 12.9°C, and annual average maximum and minimum temperatures were 42°C and −17.4°C. The total duration of daylight hours was 2,196 h yr^−1^, and the region has a frost-free period of 220 days yr^−1^, with annual mean precipitation of 550 mm yr^−1^; over 70% of the precipitation occurs between July and September. The top 1.2 m of soil at the research site is Eum-Orthrosols (Chinese Soil Taxonomy), with a mean bulk density = 1.37 g cm^−3^; average field water holding capacity = 23.4%; permanent wilting point = 7.3%; available (N) of 41.3 mg kg^−1^; available (P) of 8.56 mg kg^−1^; and available (K) of 100 mg kg^−1^. The organic matter content and pH of the first 0–20 cm of topsoil were 11.29 g kg^−1^ and 7.73, respectively.

### Experimental design and field management

The field experiment was carried out under the three large-scale waterproof sheds. The inside shed size was 32 m (length) × 15 m (width) × 3 m (height). The sheds had a transparent plastic-covered roof and four open sides. The remote control mobile water proof sheds which can move with the help of electricity, were used to control natural precipitation on rainy days. The experimental trial consisted of two planting patterns (RF: ridge furrow rainwater harvesting system, TF: conventional flat planting) with three simulated rainfall intensity (1: 275, 2: 200, 3: 125 mm) and three deficit irrigation levels (150, 75, 0 mm) in a randomized complete block design with three replication. The total of eighteen treatment combinations were coded as RF1_150_, RF1_75_, RF1_0_, RF2_150_, RF2_75_, RF2_0_, RF3_150_, RF3_75_, RF3_0_, TF1_150_, TF1_75_, TF1_0_, TF2_150_, TF2_75_, TF2_0_, TF3_150_, TF3_75_, and TF3_0_. The three simulated precipitation concentrations were applied at different winter wheat growth stages (Table 1). Half of the deficit irrigation was applied on December 12, 2015 and December 15, 2016 (tillering stage), and the other half was applied on March 28, 2016 and March 25, 2017 (flowering stage) with a precise water meter. The deficit irrigation quantity for 150 and 75 mm was determined according to the real irrigation area: under the RF system the irrigation area of two furrows was 3.78 m^2^ (1.2 × 3.15 m) and the irrigation amount of two furrows was 0.57 and 0.28 m^3^; while the irrigation area for TF planting was 6.3 m^2^ (2.0 × 3.15 m) and the irrigation amount was 0.95 and 0.47 m^3^ under 150 and 75 mm.

**Table 1:** Partition of rainfall simulation during winter wheat-growing seasons.

**Growth stages**	**Rainfall events**	**Rainfall duration**	**Daily rainfall distribution (mm)**		
			**125 mm**	**200 mm**	**275 mm**
Seedling	2	28–29 October	25	32	40
		24–25 November	13	22	30
Tillering	2	18–19 December	4	5	4
		22–23 January	3	5	6
Jointing	1	26–27 February	5	10	12
Flowering	1	20–21 March	15	24	43
Grain filling	3	9–10 April	15	30	25
		26–27 April	15	22	25
		10–11 May	15	25	45
Ripening	1	23–24 May	15	25	45

The RF planting pattern using ridge height of 15 cm with ridge and furrow widths of 40:60 cm; plastic film that had a thickness of 0.008 mm (Tianshui Tianbao Plastic Industry Ltd, Gansu, and China) were covered all the ridges. Four rows of winter wheat were planted in furrows (Figure 2). The length and width of each cemented pond plot was 3.15 × 2.0 m with 3 m depth and plots were split by 17 cm thick concrete walls to avoid exchange of soil water contents between plots. The ridge surface was covered by plastic film with the edges hidden 4–5 cm deep in the soil. During each crop growth season weeds were controlled manually.

**Figure 1:**
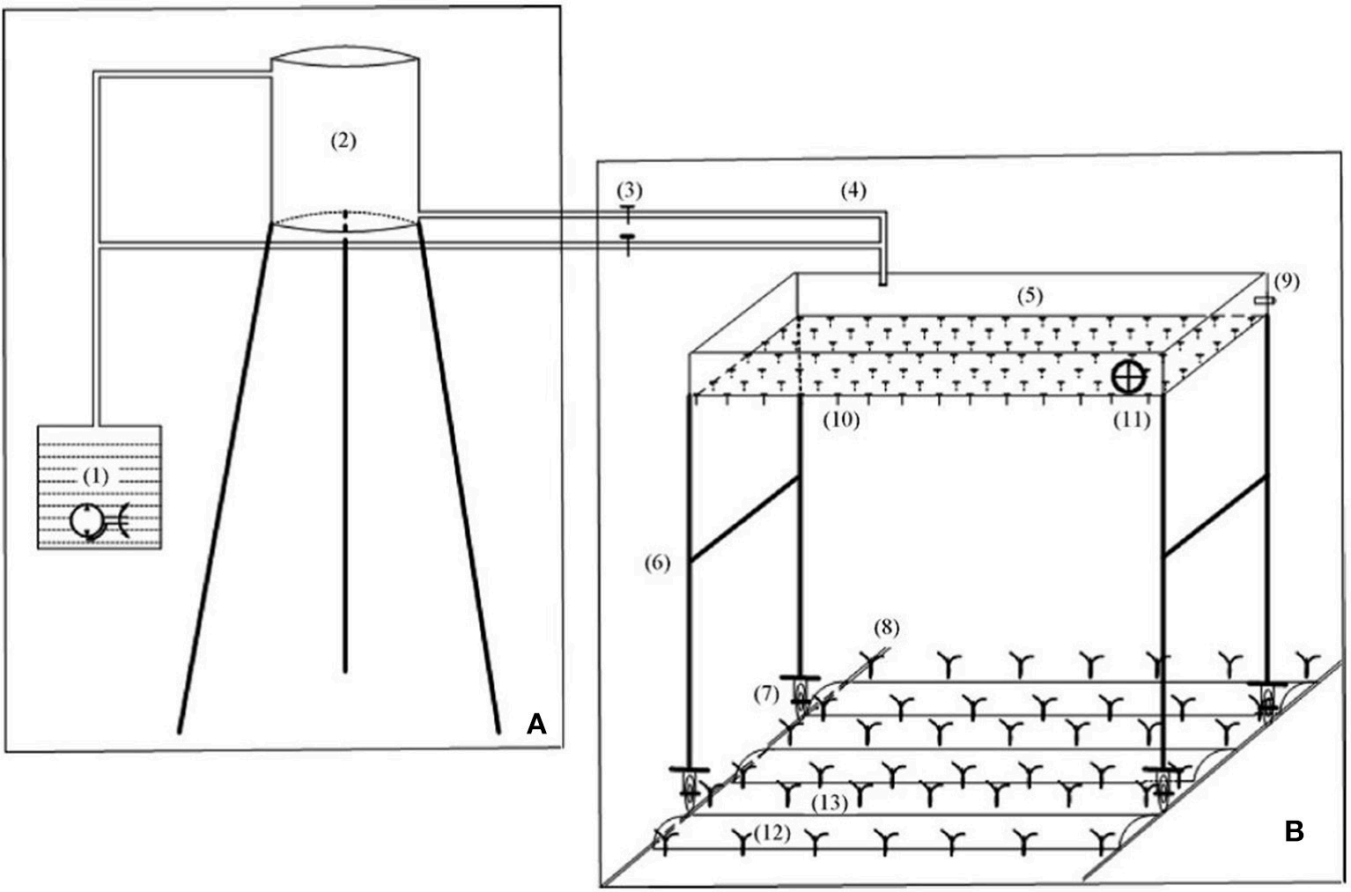
A schematic diagram showing simulator rainfall combined with ridge-furrow rainwater harvesting system under different deficit irrigation levels. **(A)** Water supply part; **(B)** rainfall simulation part; (1) water source with a pump; (2) water storage tank; (3) valves of the water pipes; (4) admitting pipe; (5) water tank made of iron sheet; (6) detachable support for the water tank; (7) pulley; (8) removable orbit; (9) surplus water drainage pipe; (10) syringe pinheads; (11) oscillating device; (12) ridges covered with plastic film; (13) planting furrows.

Winter wheat (Xinong 979) was sowed at the rate of 2,250,000 seeds per ha. The seed were planted on October 15 in 2015 and on October 10 in 2016 with an inter-row space of 20 cm. Winter wheat was harvested on June 2 in 2016 and on May 27 in 2017. All of the nitrogen and phosphorus were apply in the form of urea and diammonium phosphate (DAP) at the rates of 225 and 75 kg ha^−1^, at the time of sowing. The rainfall level partitioning was derived from the spatial and temporal characteristics of the rainfall distribution in the semi-arid regions of northern China over the past 48-years period (1966–2014). In the rainfall simulation, three total seasonal rainfalls, 125, 200, and 275 mm, corresponded to light, moderate, and heavy simulated rain levels. In this study, a precipitation simulator was used to provide the crop water requirements and no natural precipitation allowed during the winter wheat growing season (Figure 1). The precipitation simulator was used according to methods in previous studies (Ren et al., 2008). A complete detail of the precipitation determination is given in Table 1. In this simulated precipitation study, the application amount of rainfall events were not absolutely realistic under field conditions but were realistically close to it.

**Figure 2:**
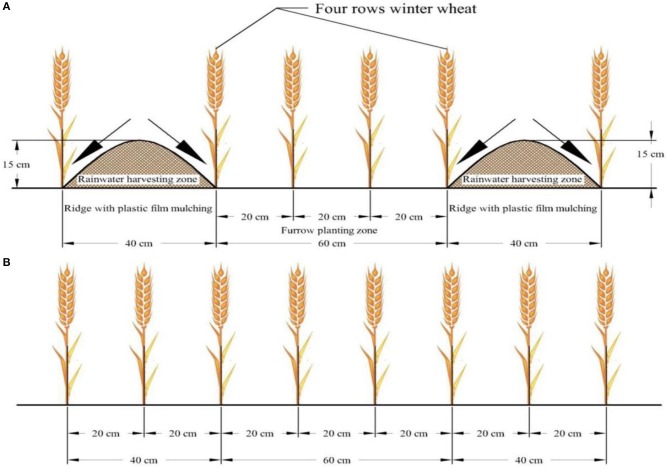
A Schematic diagram of the field layout showing **(A)** RF system (Ridge covered with plastic film mulch) and **(B)** TF (traditional flat planting).

### Sampling and measurement

Soil water content was calculated gravimetrically (g g^−1^) in the furrow to a depth of 200 cm at 20 cm intervals, at multiple time points (0, 30, 60, 90, 120, 150, 180, and 210 days after planting, DAP), from 2015 to 2017. An access tube made of seamless aluminum alloy (50 cm internal diameter) was fixed in the middle of each furrow for soil water content measurements. Soil water content in 0–200 cm soil layers at 20 cm was estimated using a TDR meter (Time-Domain Reflectometry, Trase system, Soil Moisture Equipment Corp., Germany).

Soil water storage was determined by the following equation (Wu et al., 2015):

(1) $$\begin{array}{r} SWS=\overset{n}{\underset{i}{\sum }}c_{i}\times \rho_{i}\times h_{i}/10 \end{array}$$

Where *SWS* (mm) is the soil water storage; *c*_*i*_ is the gravimetric water content (%); *p*_*i*_ (g cm^−3^) is the bulk density; *h*_*i*_ (cm) is the soil depth; *n* is the number of soil layers, *i* = 1, 2,……..20 etc.

The evapotranspiration (ET) rate was measured on a seasonal estimation basis, and was determined using the following equation (Ren et al., 2008; Wu et al., 2015):

(2) $$\begin{array}{r} \text{ET}=\text{P}+\text{I}+\text{C}+\left(\text{S}{\text{W}}_{1}-\text{S}{\text{W}}_{2}\right)-\text{D}-\text{R} \end{array}$$

where P (mm) is the total rainfall; I (mm) is the deficit irrigation; C is the upward flow into the root zone; SW_1_ (mm) is the soil moisture contents at planting time, SW_2_ is the soil moisture contents at maturity stage, and both SW_1_ and SW_2_ in the RF system have average soil moisture contents in the middle of the furrow and ridge; D is the downward drainage; and R is the surface runoff. The groundwater table remains at the depth of 80 m so the upward flow into the root was insignificant. The trial field was flat so runoff was never observed, and the drainage was concerned to be insignificant over depth of 200 cm.

The water use efficiency (WUE), rainfall irrigation water use efficiency (RIWUE) and rainfall irrigation water productivity (RIWP) were determined using Equations (3–5) (Payero et al., 2008).

(3) $$\begin{array}{r} \text{WUE}=\text{Y}/\text{ET} \end{array}$$

(4) $$\begin{array}{r} \text{RIWUE}=\text{Y}/\text{R}+\text{I} \end{array}$$

(5) $$\begin{array}{r} \text{RIWP}={\text{Y}}_{1}-{\text{Y}}_{2}/\text{R}+\text{I} \end{array}$$

Where Y is the grain yield; R is the simulated rainfall; I is the deficit irrigation; Y_1_ is the seed yield of irrigated plot; and Y_2_ is the seed yield of an un-irrigated plot.

Five winter wheat plants were randomly selected from each plot at tillering, re-wintering, jointing, flowering, and maturity time in the two trial years. The total dry matter of roots and shoots from these five plants at each stage was find out after oven drying at 105°C for 1 h and then at 70°C until a stable weight was reached. At final harvest, four rows of winter wheat were hand harvested from the center of each treatment, and the grain and biomass yields were calculated based on a moisture content of 12% for the total land area used, including the combined area of the ridges and furrows.

An economic benefit for each plot was determined using the following equations (Zhang et al., 2017).

(6) $$\begin{array}{r} \text{OV}={\text{Y}}_{\text{g}}\times {\text{P}}_{\text{g}}+{\text{Y}}_{\text{b}}\times {\text{P}}_{\text{b}} \end{array}$$

(7) $$\begin{array}{r} \text{IV}=\text{LC}+\text{PMC}+\text{MCC}+\text{SFC}+\text{WC} \end{array}$$

(8) $$\begin{array}{r} \text{O}/\text{I}=\text{OV}/\text{IV} \end{array}$$

(9) $$\begin{array}{r} \text{NI}=\text{OV}-\text{IV} \end{array}$$

where OV is the output value (CNY ha^−1^); Y_g_ is the seed yield; Y_b_ is the straw yield; P_g_ and P_b_ are the local prices of winter wheat seed and straw (CNY ha^−1^), respectively; IV is the input value (CNY ha^−1^); LC: worker costs (CNY ha^−1^); PMC: plastic film costs (CNY ha^−1^); MCC: machine-cultivation costs (CNY ha^−1^); SFC: seed and fertilizer costs (CNY ha^−1^); WC: water costs (CNY ha^−1^); IV: input value (CNY ha^−1^); OV: output value (CNY ha^−1^); O/I: output/input; and NI: net profits (CNY ha^−1^).

One hundred spikes that anthesis on the same day were selected and labeled in each treatment. Six labeled spikes from each treatment were selected at 3-day gaps from anthesis to maturity. The seeds on each spike were separated into superior and inferior according to Jiang et al. (2003). To obtain a constant weight seeds were oven dried at 70°C and then weighed.

The grain-filling process was fitted using the equation of Richards's (1959) and Zhu et al. (1988):

(10) $$\begin{array}{r} G=\frac{AkBe^{-kt}}{{\left(1+Be^{-kt}\right)}^{\left(N+1\right)/N}} \end{array}$$

Where *A* is the final kernel weight (mg); *t* is the time after flowering; and *B, k*, and *N* are the coefficients determined by regression.

The active grain-filling duration was defined as the time when W was between 5% (t_1_) and 95% (t_2_) of A. The mean grain-filling rate during this time was, therefore, determined from t_1_ to t_2_.

### Statistical analysis

The analysis of variance (ANOVA) was conducted using the SPSS 13.0 program, and data from each sampling event were analyzed separately. Mean comparisons were performed using Fisher's LSD (the least significant difference) test at *P* < 0.05. The differences in grain-filling rate between the two study years were not significant (*F* < 0.05). Therefore, the data from 2015 to 2016 were combined to determine the grain-filling rate.

## Results

### Soil water storage (SWS)

**Figure 3:**
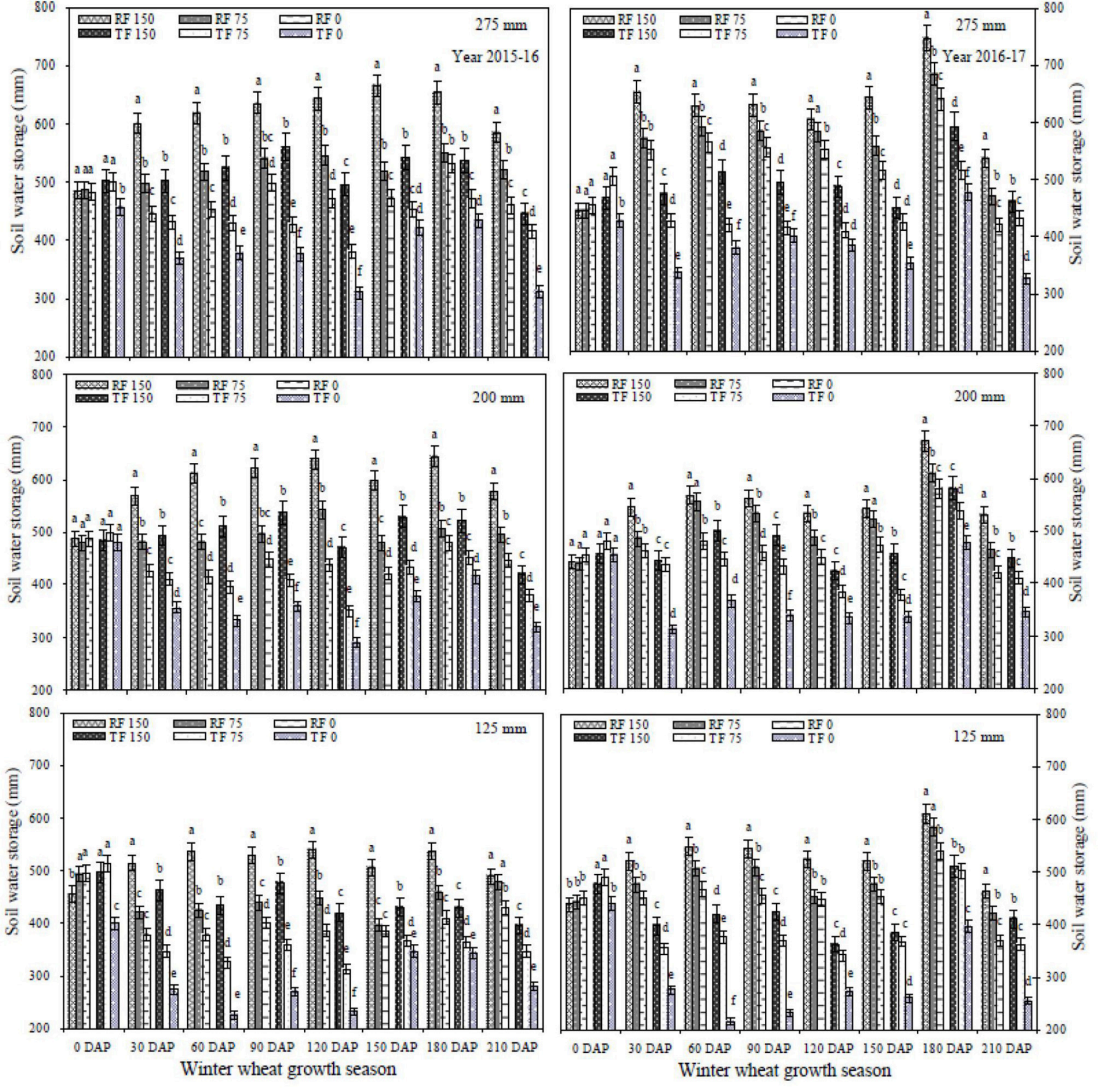
Variations in soil water storage in the soil water content dynamics in 0–200 cm layers with different treatments at different days after planting during 2015–2017. RF_150_: ridges covered with plastic film mulch and 150 mm deficit irrigation; RF_75_: ridges covered with plastic film mulch and 75 mm deficit irrigation; RF_0_: ridges covered with plastic film mulch and 0 mm deficit irrigation; TF_150_: traditional flat planting and 150 mm deficit irrigation; TF_75_: traditional flat planting and 75 mm deficit irrigation; TF_0_: traditional flat planting and 0 mm deficit irrigation. Three different simulated rainfall intensity 275, 200, and 125 mm were used. Different lowercase letters indicate significant differences at *P* < 0.05, and the vertical bars represent the standard error of the mean (*n* = 3).

In 2 successive years, the SWS profiles in the 0–2 m soil layers were determined frequently under different treatments throughout the entire growth season of winter wheat (Figure 3). At the sowing stage (0 DAP) in study years (2015–16 and 2016–17), the differences among treatments were not significant and different treatments had very similar SWS. The SWS content changed with simulated precipitation events, deficit irrigation level, planting pattern, and DAP in both study years. The soil moisture utilization rate increased as plants developed, but using the RF system under simulated rainfall and deficit irrigation reduced water stress and supplied soil water contents during critical growth stages compared to the TF planting pattern. However, the variation in SWS from 0 to 200 cm between R1_150_, R1_75_, R2_150_, R2_75_, TF1_150_, and TF2_150_ was not significant between 0 and 30 DAP. From 30 to 60 DAP, the SWS enhanced progressively with all the treatments except 75 and 0 mm deficit irrigation under the TF planting pattern. Compared with the TF2_0_ treatment, mean SWS over two years for the R2_150_ treatment had significantly increased (40.6%) at 60 DAP. SWS increased in each treatment from 60 to 120 DAP in both study years, compared to 30 DAP. From 60 to 90 DAP mean SWS in the 0–2 m depth was significantly higher (29.1%) in plots with the RF system compared to TF planting plots. However, from 120 to 150 DAP, SWS gradually decreased in all treatments (except the R1_150_treatment) compared to 90 DAP during both years. From 150 to 180 DAP, SWS increased in each treatment compared to 150 DAP; mean SWS was significantly greater (23.4%) under the RF system than under TF planting. Compared with TF2_150_ treatment, mean SWS over two years in the R2_150_ treatment had significantly increased (19.6%) at 180 DAP. The SWS capacity in the 0–2 m soil depth under the RF system significantly increased with increasing simulated rainfall intensity and deficit irrigation levels compared to TF planting during all growth stages of winter wheat. An increase in simulated rainfall level from 200 to 275 mm caused SWS to increase gradually at both 75 and 150 mm deficit irrigation levels; however there was slight declines at 210 DAP under both planting patterns.

### Evapotranspiration

There were obvious deviations in evapotranspiration (ET) rate between the different treatments during the winter wheat growing seasons (**Tables 3, 4**). Winter wheat growth and development was faster during the middle growth stages, with higher consumption of simulated precipitation and deficit irrigation water, and as a result, higher crop evapotranspiration. The TF planting pattern under different deficit irrigation levels with simulated rainfall conditions led to higher ET rates than the RF system. The mean over two years of ET showed that the RF system improved soil moisture content and reduced evaporation; the ET rate was 109 mm (41%) lower than that of TF planting pattern. Average ET over two years indicated that ET rates of RF1_150_, RF1_75_, and RF1_0_ were significantly (*P* < 0.05) reduced by 128 mm (28%), 123 mm (28%), and 95 mm (24%), respectively, compared to TF1_150_, TF1_75_, and TF1_0_, respectively. Compared with TF2_150_, TF2_75_, and TF2_0_ treatments, mean ET rates over 2 years of RF2_150_, RF2_75_, and RF2_0_ treatments were significantly (*P* < 0.05) reduced by 127 mm (33%), 130 mm (34%), and 96 mm (29%), respectively. The ET rates were significantly reduced by 114 mm (32%) for RF3_150_, 130 mm (37%) for RF3_75_, and 35 mm (15%) for RF3_0_, compared with TF3_150_, TF3_75_, and TF3_0_ treatments, respectively.

### Grain filling process

Simulated rainfall with different planting patterns significantly influenced the maximum grain weight and grain-filling process of wheat crop under deficit irrigations. Rising the simulated rainfall level from 125 to 200 mm under different planting patterns and deficit irrigations, caused significant increases in the maximum kernel weights and maximum and mean grain-filling rates for superior and inferior grains; however, when simulated rainfall increased from 200 to 275 mm, there were no significant influence on grain-filling rates under different planting patterns (Table 2). The RF planting model also significantly influenced the grain-filling rates compared to TF planting. At 125 mm of precipitation, the RF_150_ treatment significantly improved the maximum grain weights and the maximum and mean seed-filling rates of the superior and inferior grains compared to TF_150_ treatments. At 200 mm precipitation, the RF_150_ and TF_150_treatments significantly improved the maximum grain weights and the maximum and mean seed filling rates of the superior grains, compared to RF_0_ and TF_0_ treatments; however, the RF system had no significant influences on these variables for the inferior grains. At 275 mm simulated precipitation, there were no significant differences between the RF and TF planting models for these parameters under 150 and 75 mm deficit irrigation; however, significant variation was recorded between 0 mm with 75 and 150 mm irrigation for maximum kernel weights, and maximum and mean grain-filling rates for the superior and inferior grains at 275 mm simulated precipitation conditions under both planting patterns.

**Table 2:** Effects of different planting patterns, simulated rainfall and deficit irrigation levels on grain-filling characteristics of winter wheat in 2015–2016.

	**Simulated rainfall (mm)**	**Treatments**	**W_max_(mg)**	**G_mean_ (mg grain^−1^ d^−1^)**	**G_max_ (mg grain^−1^ d^−1^)**
Superior grain	275	RF_150_	50.1a	1.47a	3.21b
		RF_75_	49.8a	1.44a	3.19c
		RF_0_	47.2b	1.35c	2.64f
		TF_150_	49.1a	1.44a	3.18c
		TF_75_	47.6b	1.40b	3.09c
		TF_0_	46.1b	1.31d	2.51g
	200	RF_150_	48.9a	1.45a	3.51a
		RF_75_	46.1b	1.39c	3.23b
		RF_0_	43.5c	1.29e	2.82e
		TF_150_	46.2b	1.41b	3.30b
		TF_75_	44.1c	1.31d	3.01d
		TF_0_	42.5d	1.22e	2.52g
	125	RF_150_	42.5d	1.27e	3.02d
		RF_75_	41.3d	1.15f	2.71e
		RF_0_	39.8e	1.01g	2.30h
		TF_150_	41.4d	1.19f	2.62f
		TF_75_	41.1d	1.08f	2.51g
		TF_0_	39.2e	0.88h	2.09i
Inferior grain	275	RF_150_	44.1a	0.85a	1.12b
		RF_75_	43.7a	0.81a	1.11b
		RF_0_	42.6ab	0.73b	0.99c
		TF_150_	43.7a	0.81a	1.02b
		TF_75_	42.6ab	0.78b	1.04b
		TF_0_	42.1ab	0.70c	0.88c
	200	RF_150_	43.5a	0.75b	1.31a
		RF_75_	42.4ab	0.67c	1.21a
		RF_0_	41.7b	0.56d	1.07b
		TF_150_	42.8ab	0.69c	1.13b
		TF_75_	41.7b	0.59d	0.98c
		TF_0_	40.1b	0.53e	0.85d
	125	RF_150_	39.1c	0.40f	0.96c
		RF_75_	38.0c	0.34g	0.87d
		RF_0_	36.0d	0.24h	0.64e
		TF_150_	37.2d	0.38g	0.77d
		TF_75_	36.1d	0.28h	0.56e
		TF_0_	34.3e	0.21h	0.47f

*Mean values within a column and for the same grain type followed by different letters are significantly differences at P ≤ 0.05 levels. RF_150_: Ridges covered with plastic film mulch and 150 mm deficit irrigation; RF_75_: ridges covered with plastic film mulch and 75 mm deficit irrigation; RF_0_: ridges covered with plastic film mulch and 0 mm deficit irrigation; TF_150_: traditional flat planting with 150 mm deficit irrigation; TF_75_: traditional flat planting with 75 mm deficit irrigation; TF_0_: traditional flat planting with 0 mm deficit irrigation. W_max_: the final maximum grain weight; G_max_: maximum grain-filling rates; G_mean_: mean grain-filling rates*.

### Total dry matter g per plant and biomass yield

Under the RF system in both study years, total biomass (shoot + root) g per plant and biomass yield were significantly maximum compared to the TF planting model under simulated rainwater concentrations with deficit irrigation. The RF system significantly (*P* < 0.05) increased the biomass yield to 1.5 t ha^−1^ (14%) compared to the TF planting model. The total biomass per plant increased slowly in the early growth stages (tillering and re-wintering), quickly in the middle growth stages (jointing and flowering), and reached a maximum value during physiological maturity; there were no significant differences with rainfall of more than 200 mm under both planting patterns. There were no significant variations between RF1_150_ and RF2_150_ treatments and produced a maximum total dry matter per plant during entire growth stages of winter wheat crop. Average biomass yield was significantly increased in RF1_150_, RF1_75_, TF1_150_, TF1_75_, and RF1_0_ treatments by 59, 51, 46, 41, and 22%, respectively, compared with the TF3_0_ treatment. The mean biomass yields for RF2_150_, TF2_150_, RF2_75_, TF2_75_, and RF2_0_ were significantly (*P* < 0.05) increased by 143, 119, 111, 81, and 43%, respectively, compared to TF2_0_. Compared with TF3_0_, average biomass yield for RF3_150_, TF3_150_, RF3_75_, TF3_75_, and RF3_0_ treatments were significantly increased by 146, 129, 96, 85, and 23%, respectively. Increasing the precipitation from 200 to 275 mm under both planting patterns caused biomass yield to increase but there were no significant differences under 150 mm deficit irrigation.

### Water use efficiency and grain yield

The water use efficiency (WUE) and grain yield of wheat varied significantly under simulated rainfall conditions with different planting patterns and deficit irrigations in both study years (Tables 3, 4). The WUE and grain yield tended to increase with the simulated rainfall and deficit irrigations levels, but there were no significant differences when the simulated precipitation was above 200 mm under both planting patterns in both years. The RF system under deficit irrigations with simulated rainfall conditions led to higher grain yield than TF planting. The mean of two years data showed that the RF system increased SWS and reduced ET; the grain yield was 0.82 t ha^−1^ (19%) higher than TF. The mean over two years of grain yield indicated that RF1_150_, RF1_75_, and RF1_0_ were significantly increased by 0.73 t ha^−1^ (10.61%), 0.77 t ha^−1^ (12.58%), and 0.88 t ha^−1^ (20.85%), respectively, compared to TF1_150_, TF1_75_, and TF1_0_, respectively. Compared with TF2_150_, TF2_75_, and TF2_0_ treatments average grain yield of RF2_150_, RF2_75_, and RF2_0_ treatments were significantly increased by 1.19 t ha^−1^ (18.90%), 1.15 t ha^−1^ (22.50%), and 1.13 t ha^−1^ (42.13%), respectively. Average grain yield was significantly increased by 0.59 t ha^−1^ (14.29%) in RF3_150_, 0.29 t ha^−1^ (8.94%) in RF3_75_, and 0.77 t ha^−1^ (53.50%) in RF3_0_, compared with TF3_150_, TF3_75_, and TF3_0_ treatments, respectively.

**Table 3:** Effects of different treatments^a^ on biomass at harvest (t ha^−1^), grain yield (t ha^−1^), evapotranspiration (ET, mm), and water use efficiency (WUE, kg ha^−1^ mm^−1^) of winter wheat in 2015–2016^b^.

**Treatments**	**Biomass (t ha^−1^)**	**Grain yield (t ha^−1^)**	**ET (mm)**	**WUE (kg ha^−1^ mm^−1^)**
RF1_150_	16.44 ± 0.91a	7.53 ± 0.19a	323.92 ± 36.3d	23.26 ± 0.8a
RF1_75_	15.60 ± 0.50ab	7.07 ± 0.58a	314.22 ± 41.4d	22.51 ± 0.6a
RF1_0_	13.60 ± 0.47c	4.47 ± 0.68c	297.08 ± 33.5e	15.05 ± 0.9b
TF1_150_	15.36 ± 0.34ab	6.86 ± 0.46b	482.32 ± 45.3a	14.22 ± 1.2b
TF1_75_	15.10 ± 0.23b	6.42 ± 0.18b	463.05 ± 39.6ab	13.85 ± 0.9c
TF1_0_	11.40 ± 0.17d	3.92 ± 0.13d	421.17 ± 47.1c	9.30 ± 0.7d
RF2_150_	16.22 ± 0.38a	7.40 ± 0.58a	260.62 ± 37.4c	28.39 ± 1.0a
RF2_75_	14.25 ± 0.42b	6.53 ± 0.51b	259.60 ± 43.2c	25.17 ± 1.1b
RF2_0_	10.71 ± 0.68d	4.15 ± 0.68d	243.64 ± 37.9cd	17.05 ± 0.5bc
TF2_150_	14.52 ± 0.19b	6.26 ± 0.32b	414.52 ± 35.2a	15.10 ± 1.1c
TF2_75_	13.21 ± 0.52c	5.62 ± 0.31c	410.91 ± 41.6a	13.68 ± 0.6d
TF2_0_	8.05 ± 0.33e	3.19 ± 0.14e	360.26 ± 38.1b	8.84 ± 0.7e
RF3_150_	12.67 ± 0.55a	5.42 ± 0.38a	241.00 ± 32.1b	22.50 ± 0.8a
RF3_75_	10.65 ± 0.59b	4.15 ± 0.52b	215.16 ± 39.8c	19.30 ± 0.7b
RF3_0_	6.06 ± 0.24d	2.41 ± 0.18d	190.43 ± 33.8cd	12.64 ± 0.8c
TF3_150_	11.73 ± 0.66a	4.47 ± 0.37b	376.48 ± 43.8a	11.88 ± 0.8c
TF3_75_	9.94 ± 0.46c	3.80 ± 0.26c	367.05 ± 37.5a	10.36 ± 0.5cd
TF3_0_	5.01 ± 0.33e	1.47 ± 0.29e	204.00 ± 33.9c	7.21 ± 0.9d

a RF1: ridges covered with plastic film mulch and 275 mm simulated rainfall; RF2: ridges covered with plastic film mulch and 200 mm simulated rainfall; RF3: ridges covered with plastic film mulch and 125 mm simulated rainfall; TF1: traditional flat planting and 275 mm simulated rainfall; TF2: traditional flat planting and 200 mm simulated rainfall; TF3: traditional flat planting and 125 mm simulated rainfall; _150_: 150 mm deficit irrigation; _75_: 75 mm deficit irrigation; _0_: 0 mm deficit irrigation;

b *Values are given as means ± standard deviations, and different lowercase letters indicate significant differences at P ≤ 0.05 levels in the same line (Duncan's multiple range test)*.

**Table 4:** Effects of different treatments^a^ on biomass at harvest (t ha^−1^), grain yield (t ha^−1^), evapotranspiration (ET, mm), and water use efficiency (WUE, kg ha^−1^ mm^−1^) of winter wheat in 2016–2017^b^.

**Treatments**	**Biomass (t ha^−1^)**	**Grain yield (t ha^−1^)**	**ET (mm)**	**WUE (kg ha^−1^ mm^−1^)**
RF1_150_	16.93 ± 0.93a	7.79 ± 0.18a	332.43 ± 38.4c	23.43 ± 0.7a
RF1_75_	16.25 ± 0.51a	6.62 ± 0.68b	325.55 ± 39.3c	20.35 ± 0.9b
RF1_0_	12.16 ± 0.57c	4.92 ± 0.67d	310.39 ± 44.1cd	15.85 ± 0.8c
TF1_150_	15.41 ± 0.38b	6.99 ± 0.59b	430.05 ± 42.2a	16.24 ± 0.9c
TF1_75_	14.56 ± 0.27b	5.74 ± 0.15c	423.42 ± 37.6a	13.55 ± 0.7d
TF1_0_	9.63 ± 0.13d	3.85 ± 0.10e	377.14 ± 48.9b	10.22 ± 0.5e
RF2_150_	16.69 ± 0.44a	7.57 ± 0.64a	260.36 ± 41.5c	29.06 ± 1.2a
RF2_75_	14.37 ± 0.39b	5.99 ± 0.52c	250.26 ± 34.3c	23.95 ± 0.9b
RF2_0_	8.71 ± 0.93d	3.44 ± 0.78e	233.78 ± 36.2d	14.73 ± 0.6d
TF2_150_	15.10 ± 0.15a	6.33 ± 0.22b	359.84 ± 34.2a	17.58 ± 1.1c
TF2_75_	11.25 ± 0.54c	4.60 ± 0.23d	357.99 ± 40.1a	12.86 ± 1.2e
TF2_0_	5.50 ± 0.29e	2.15 ± 0.10f	309.33 ± 35.3b	6.94 ± 0.8f
RF3_150_	10.08 ± 0.49a	3.94 ± 0.41a	249.63 ± 38.5b	15.79 ± 0.8a
RF3_75_	7.47 ± 0.55b	2.92 ± 0.50b	221.42 ± 33.2c	13.19 ± 0.6b
RF3_0_	5.33 ± 0.19c	1.98 ± 0.13c	204.73 ± 32.8cd	9.67 ± 0.7c
TF3_150_	9.44 ± 0.60a	3.72 ± 0.35a	341.50 ± 46.1a	10.88 ± 0.6c
TF3_75_	7.15 ± 0.41b	2.69 ± 0.23b	329.01 ± 38.2b	8.17 ± 0.7cd
TF3_0_	4.24 ± 0.32*d*	1.39 ± 0.32c	261.99 ± 35.9b	5.30 ± 0.5e

a RF1: Ridges covered with plastic film mulch and 275 mm simulated rainfall; RF2: ridges covered with plastic film mulch and 200 mm simulated rainfall; RF3: ridges covered with plastic film mulch and 125 mm simulated rainfall; TF1: traditional flat planting and 275 mm simulated rainfall; TF2: traditional flat planting and 200 mm simulated rainfall; TF3: traditional flat planting and 125 mm simulated rainfall; _150_: 150 mm deficit irrigation; _75_: 75 mm deficit irrigation; _0_: 0 mm deficit irrigation;

b *Values are given as means ± standard deviations, and different lowercase letters indicate significant differences at P ≤ 0.05 levels in the same line (Duncan's multiple range test)*.

WUE increased along with the increase in grain yield. The WUE increased by 8.10 kg mm^−1^ ha^−1^ (70.67%) under the RF system compared to TF, due to a decrease in the ET rate at the field scale. Compared with TF1_150_, TF1_75_, and TF1_0_ treatments, mean values of WUE over two years for RF1_150_, RF1_75_, and RF1_0_ treatments were significantly improved by 8.12 kg mm^−1^ ha^−1^ (53.28%), 7.73 kg mm^−1^ ha^−1^ (56.42%), and 5.69 kg mm^−1^ ha^−1^ (58.30%). Mean WUE of RF2_150_, RF2_75_, and RF2_0_ were significantly increased by 12.39 kg mm^−1^ ha^−1^ (75.80%), 11.29 kg mm^−1^ ha^−1^ (85.08%), and 8.00 kg mm^−1^ ha^−1^ (101.39%), compared to TF2_150_, TF2_75_, and TF2_0_, respectively. Average WUE was significantly increased in RF3_150_, RF3_75_, and RF3_0_, treatments by 7.77 kg mm^−1^ ha^−1^ (68.23%), 6.98 kg mm^−1^ ha^−1^ (75.34%), and 4.90 kg mm^−1^ ha^−1^ (78.34%), respectively, compared with TF3_150_, TF3_75_, and TF3_0_ treatments.

### RIWUE and RIWP

**Figure 4:**
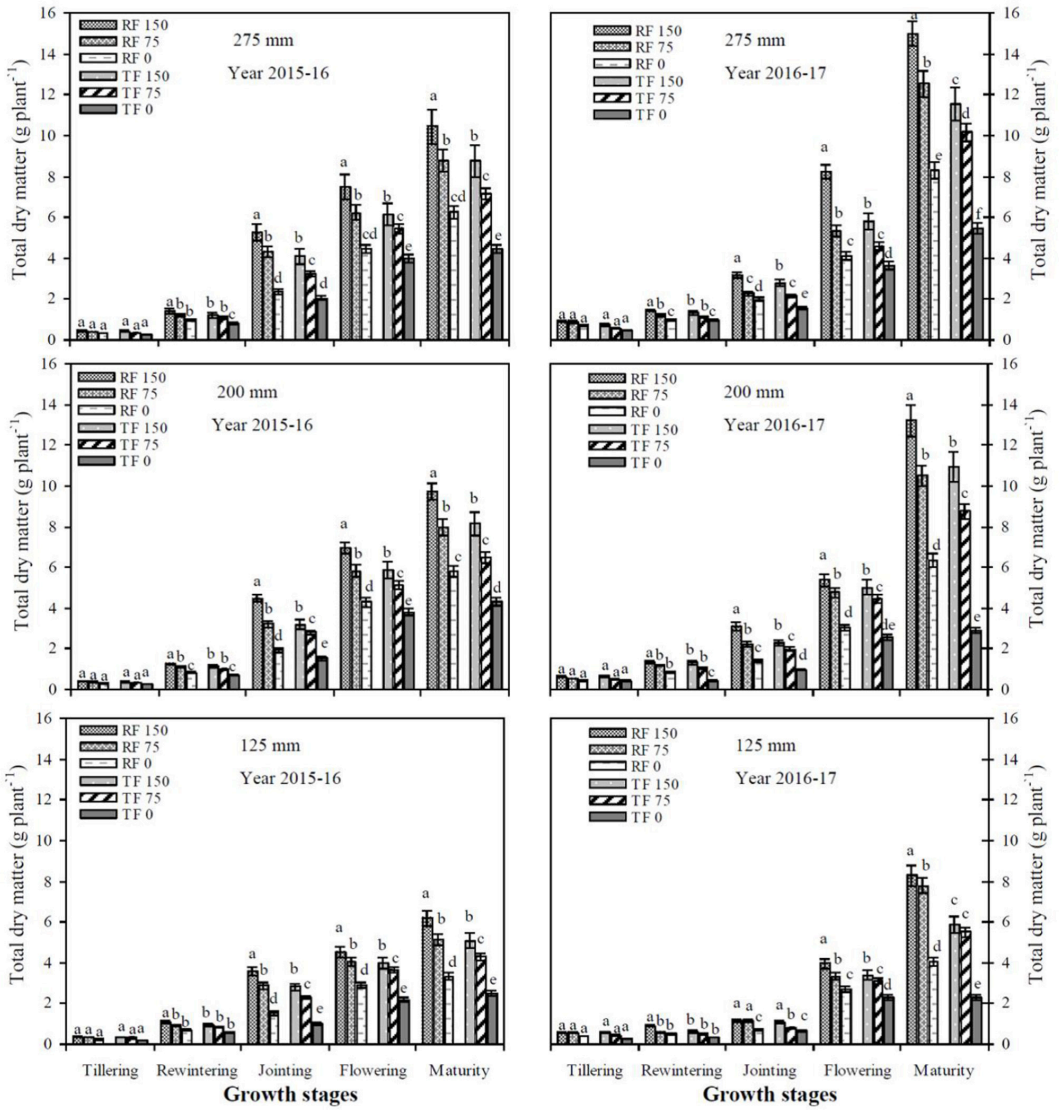
Effects of planting patterns and deficit irrigation on total dry matter accumulation (shoot + root) under simulated rainfall conditions during the winter wheat growing seasons in 2015–2017. RF_150_: ridges covered with plastic film mulch and 150 mm deficit irrigation; RF_75_: ridges covered with plastic film mulch and 75 mm deficit irrigation; RF_0_: ridges covered with plastic film mulch and 0 mm deficit irrigation; TF_150_: traditional flat planting and 150 mm deficit irrigation; TF_75_: traditional flat planting and 75 mm deficit irrigation; TF_0_: traditional flat planting and 0 mm deficit irrigation. Three different simulated rainfall intensity 275, 200, and 125 mm were used. Different lowercase letters indicate significant differences at *P* < 0.05, and the vertical bars represent the ± the standard error of the mean (*n* = 3).

**Figure 5:**
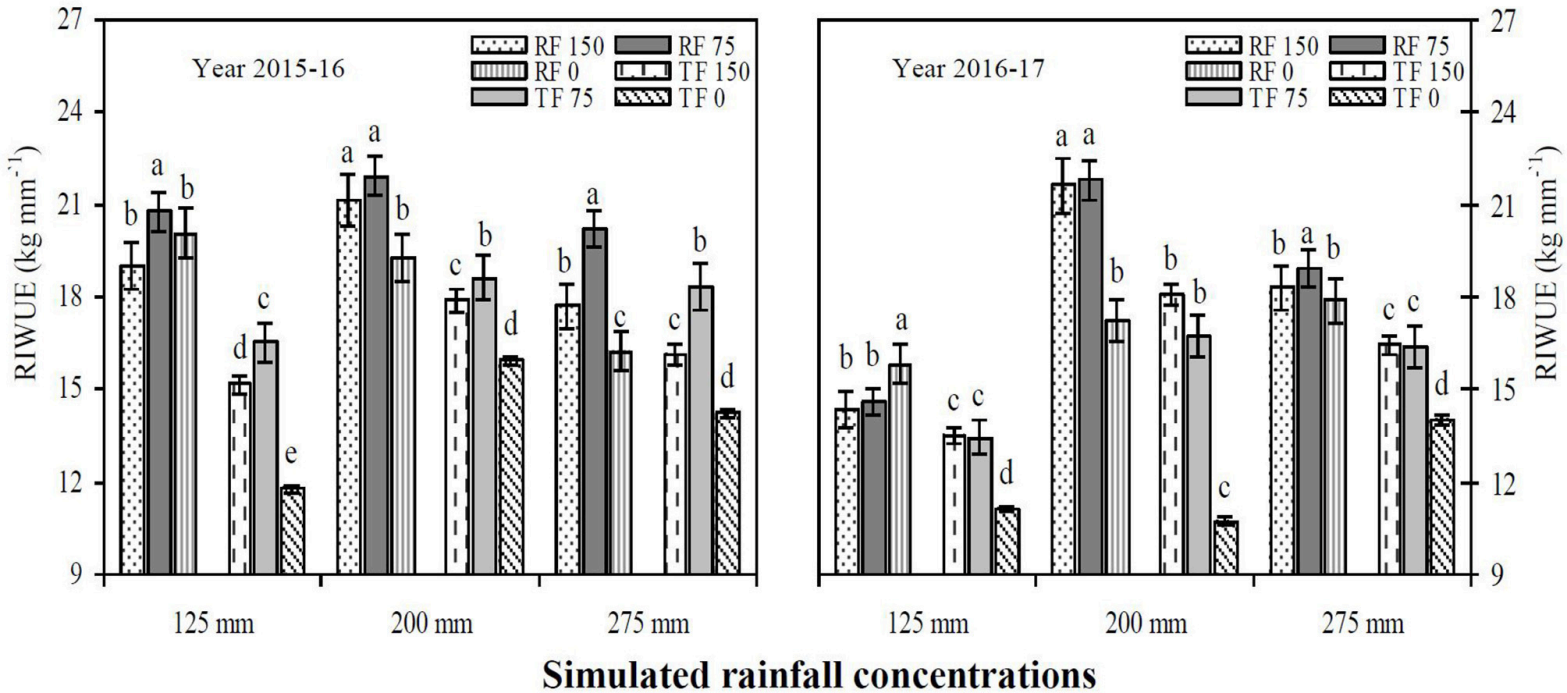
Rainfall irrigation water use efficiency (RIWUE) in 2015–16 and 2016–17. RF_150_: ridges covered with plastic film mulch and 150 mm deficit irrigation; RF_75_: ridges covered with plastic film mulch and 75 mm deficit irrigation; RF_0_: ridges covered with plastic film mulch and 0 mm deficit irrigation; TF_150_: traditional flat planting and 150 mm deficit irrigation; TF_75_: traditional flat planting and 75 mm deficit irrigation; TF_0_: traditional flat planting and 0 mm deficit irrigation. Three different simulated rainfall intensity 275, 200, and 125 mm were used. Different lowercase letters indicate significant differences at *P* < 0.05, and the error bars represent the standard error of the mean (*n* = 3).

**Figure 6:**
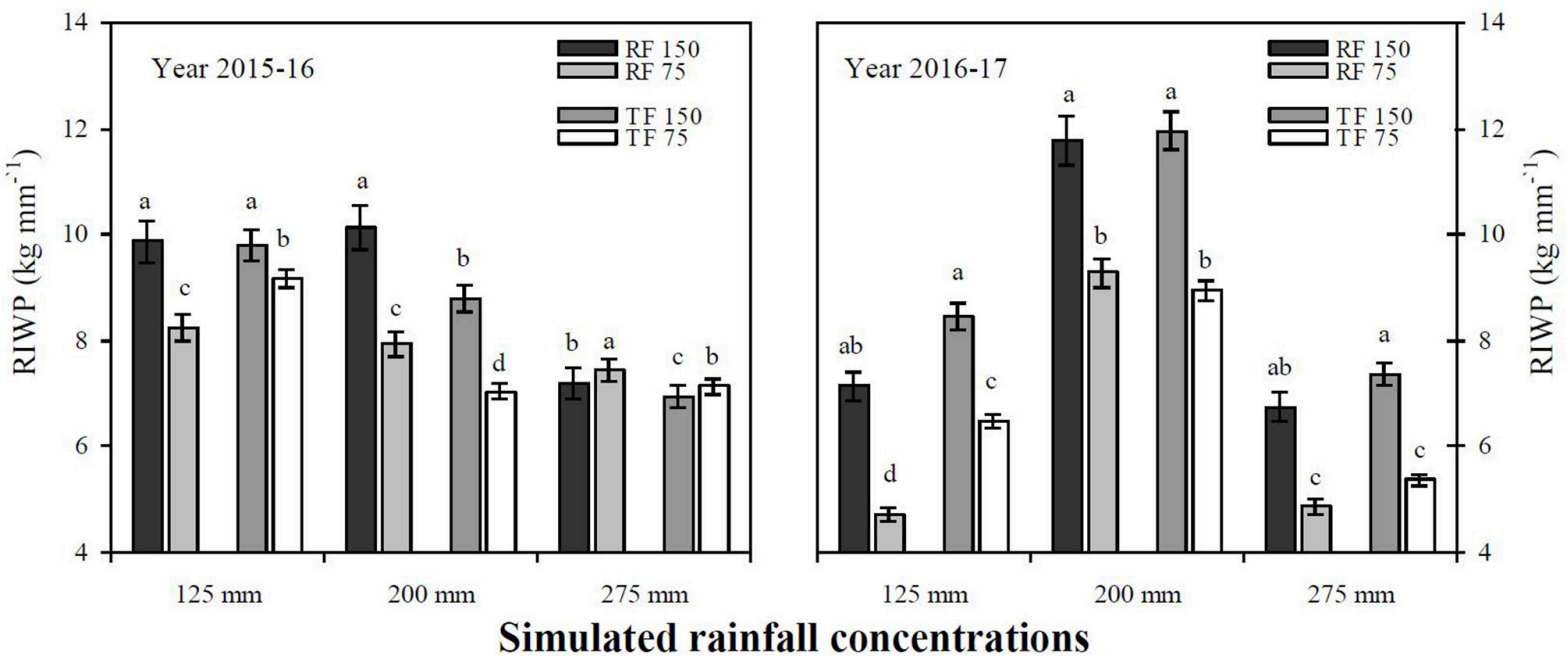
Rainfall irrigation water productivity (RIWP) in 2015–16 and 2016–17. RF_150_: ridges covered with plastic film mulch and 150 mm deficit irrigation; RF_75_: ridges covered with plastic film mulch and 75 mm deficit irrigation; TF_150_: traditional flat planting and 150 mm deficit irrigation; TF_75_: traditional flat planting and 75 mm deficit irrigation. Three different simulated rainfall intensity 275, 200, and 125 mm were used. Different lowercase letters indicate significant differences at *P* < 0.05, and the error bars represent the standard error of the mean (*n* = 3).

The RF system with deficit irrigation under simulated precipitation significantly improved the RIWUE and RIWP of winter wheat compared to the TF planting pattern during both study years (Figures 4, 5). The average mean of two years showed that the RF system significantly improved RIWUE 3.44 kg mm^−1^ (22.50%) compared to the TF planting pattern. In addition, the RF2_150_ treatment achieved the maximum grain yield by utilizing less irrigation and rainfall water. As a result, the RIWUE with RF2_150_ treatment was 3.35 and 5.09 kg mm^−1^ higher than RF1_150_ and TF1_150_ treatments, respectively (Figure 5). The RIWP shows the capacity of winter wheat grain yield to increase through the addition of simulated rainfall and irrigation water. The RF2_150_ treatment in 2015–16 and the RF2_150_ and TF2_150_ treatments in 2016–17 used simulated rainfall efficiently, and deficit irrigation was decreased by half (Figure 6). Compared with RF1_150_, RF1_75_, TF1_150_, and TF1_75_ treatments average RIWP over both years for RF2_150_, TF2_150_, RF2_75_, and TF2_75_ treatments were significantly (*P* < 0.05) improved by 3.98 kg mm^−1^ (57.00%), 3.22 kg mm^−1^ (44.97%), 2.45 kg mm^−1^ (39.81%), and 1.73 kg mm^−1^ (27.61%), respectively. Mean RIWP was significantly increased in RF2_150_, RF2_75_, TF2_150_, and TF2_75_ treatments by 2.45 kg mm^−1^ (28.77%), 2.13 kg mm^−1^ (32.96%), 1.22 kg mm^−1^ (13.40%), and 0.16 kg mm^−1^ (2.02%), respectively, compared with RF3_150_, RF3_75_, TF3_150_, and TF3_75_ treatments, respectively.

### Economic benefit

There were clear variations in the total input costs of the different treatments due to the costs of plastic film, labor, and water (Table 5). The mean of two years input cost was order as follows: RF1_150_ > RF1_75_ ≈ RF2_150_ > RF1_0_ ≈ RF2_75_ ≈ RF3_150_ > RF2_0_ ≈ RF3_75_ > RF3_0_ > TF1_150_ > TF1_75_ ≈ TF2_150_ > TF1_0_ ≈ TF2_75_ ≈ TF3_150_ > TF2_0_ ≈ TF3_75_ > TF3_0_. However, the RF2_150_ treatment had a significantly positive effect on the net income (12,593 CNY ha^−1^), output/ input ratio (2.65) and net income difference (10,052 CNY ha^−1^) compared to all other treatments. The net income values were in the following order: RF2_150_ > RF1_150_ > TF1_150_ > TF2_150_ > TF1_75_ > RF1_75_ > RF2_75_ > TF2_75_ > TF3_150_ > RF1_0_ > RF3_150_ > TF1_0_ > TF3_75_ > TF2_0_ > RF2_0_ > RF3_75_ > RF3_0_ > TF3_0_, which increased by 12,593, 12,530, 11,996, 11,170, 10,968, 10,719, 9,166, 8,019, 5,743, 5,258, 4,836, 4,525, 3,362, 2,647, 2,541, 2,172, −1,122, and −1,128 CNY ha^−1^, respectively.

**Table 5:** Average economic benefits [Chinese Yuan (CNY) ha^−1^] under different treatments^a^.

**Treatments**	**LC**	**PMC**	**MCC**	**SFC**	**WC**	**IV**	**OV**	**O/I**	**NI**	**NID**
RF1_150_	2,650	710	1,500	2,027	638	7,525	20,055	2.67	12,530	7,272
RF1_75_	2,650	710	1,500	2,027	525	7,312	18,031	2.46	10,719	5,461
RF1_0_	2,650	710	1,500	2,027	413	7,300	12,558	1.72	5,258	
RF2_150_	2,650	710	1,500	2,027	525	7,312	19,905	2.65	12,593	10,052
RF2_75_	2,650	710	1,500	2,027	413	7,300	16,466	2.26	9,166	6,625
RF2_0_	2,650	710	1,500	2,027	300	7,187	9,728	1.35	2,541	
RF3_150_	2,650	710	1,500	2,027	413	7,300	12,136	1.66	4,836	5,958
RF3_75_	2,650	710	1,500	2,027	300	7,187	9,360	1.30	2,172	3,294
RF3_0_	2,650	710	1,500	2,027	188	7,075	5,953	0.84	−1,122	
TF1_150_	2,650	0	1,500	2,027	638	6,815	18,811	2.76	11,996	7,471
TF1_75_	2,650	0	1,500	2,027	525	6,702	17,671	2.64	10,968	6,443
TF1_0_	2,650	0	1,500	2,027	413	6,590	11,115	1.69	4,525	
TF2_150_	2,650	0	1,500	2,027	525	6,702	17,872	2.67	11,170	8,523
TF2_75_	2,650	0	1,500	2,027	413	6,590	14,609	2.22	8,019	5,372
TF2_0_	2,650	0	1,500	2,027	300	6,477	9,124	1.41	2,647	
TF3_150_	2,650	0	1,500	2,027	413	6,590	12,332	1.87	5,743	6,871
TF3_75_	2,650	0	1,500	2,027	300	6,477	9,840	1.52	3,362	4,490
TF3_0_	2,650	0	1,500	2,027	188	6,365	5,237	0.82	−1,128	

a *RF1: ridges covered with plastic film mulch and 275 mm simulated rainfall; RF2: ridges covered with plastic film mulch and 200 mm simulated rainfall; RF3: ridges covered with plastic film mulch and 125 mm simulated rainfall; TF1: traditional flat planting and 275 mm simulated rainfall; TF2: traditional flat planting and 200 mm simulated rainfall; TF3: traditional flat planting and 125 mm simulated rainfall; _150_: 150 mm deficit irrigation; _75_: 75 mm deficit irrigation; _0_: 0 mm deficit irrigation*.

*LC: Labor costs [Chinese yuan (CNY) ha^−1^]; MC: film mulching costs (CNY ha^−1^); MCC: machine-cultivation costs (CNY ha^−1^); SFC: seed and fertilizer costs (CNY ha^−1^); WC: water costs (CNY ha^−1^); IV: input value (CNY ha^−1^); OV: output value (CNY ha^−1^); O/I: output/input; NI: net income (CNY ha^−1^); NID: net income difference (CNY ha^−1^) as compared with control. Labor cost = 80 CNY per person day^−1^; plastic film cost = 12 CNY kg^−1^; wheat grain price = 2.40 CNY kg^−1^; wheat straw price = 0.1 CNY kg^−1^*.

## Discussion

### Soil water storage and total dry matter accumulation

The RF system can increase soil water storage and prolong the period of moisture accessibility, by saving rainwater during light rainfall events, thus decreasing evaporation, and encouraging precipitation infiltration (Wang et al., 2009). Ren et al. (2008) confirmed that under the RF planting model with different precipitation concentrations (230, 340, and 440 mm), mean SWS in the depth of 2 m was 2.3, 5.2, and 4.5% higher respectively, during the summer maize seasons, compared to the flat planting model without plastic mulching. Previous research reported that the RF system has a vital role for improving WUE in dry-land farming systems (Ren et al., 2010; Chen et al., 2013). In the current research work, we found that soil moisture utilization rate increased as plants developed, but the RF system under simulated rainfall and deficit irrigation reduced water stress and supplied soil water contents during critical growth stages compared to the TF planting pattern. Compared with TF2_0_ treatment, mean SWS over two years for R2_150_ treatment significantly increased (40.6%) at 60 DAP. From 60 to 90 DAP mean SWS in the 0–2 m depth was significantly (29.1%) greater in plots with the RF system compared to plots with TF planting. However, from 120 to 150 DAP, SWS gradually decreased in all treatments (except R1_150_ treatment) compared to 90 DAP during both study years. The SWS capacity in the 0–2 m soil depth under the RF system significantly increased with increasing simulated rainfall intensity and deficit irrigation levels compared to TF planting during all growth stages of winter wheat. An increase in simulated rainfall level from 200 to 275 mm caused a gradual increase in SWS in both 75 and 150 mm levels of deficit irrigation; there was slight decreases at 210 DAP under both planting patterns. Previous studies suggest that the RF planting model combined with simulated precipitation may enhance SWS, thereby increasing moisture availability during the wheat growing season (Zhang et al., 2007; Li et al., 2013). Ren et al. (2008) studied the effect of the RF planting model on SWS with yearly rainfall between 230 and 440 mm. There were no significant influences when the precipitation goes beyond 440 mm, which is consistent with our results.

An earlier study confirmed that supplemental irrigations under the RF planting model had a significant influence on SWS: it reduced the loss of soil moisture content through evaporation, which increased total dry matter accumulation per plant, crop yields, and WUE compared to the TF planting model (Triplett and Dick, 2008; Zhou et al., 2012). The maximum improvement in biomass yield and WUE was achieved by combined use of limited irrigation under the RF planting model in semi-arid regions (Rockström et al., 2002; Li et al., 2016). We found that under the RF system significantly (*P* < 0.05) improved biomass yield by 1.5 t ha^−1^ (14%) compared to the TF planting model. The total biomass per plant increased slowly in the early growth stages (tillering and re-wintering), quickly in the middle growth stages (jointing and flowering), and reached a maximum value during physiological maturity; there were no significant differences with rainfall higher than 200 mm under both planting patterns. There were no significant variation between RF1_150_ and RF2_150_ treatments and produced a maximum total dry matter per plant during all growth stages of winter wheat. The mean biomass yield of RF2_150_, TF2_150_, RF2_75_, TF2_75_, and RF2_0_ were significantly (*P* < 0.05) increased by 143, 119, 111, 81, and 43%, compared to TF2_0_, respectively. Increasing the amount of precipitation from 200 to 275 mm under both planting patterns led to an increase in biomass yield but did not lead to significant (*P* > 0.05) differences under 150 mm deficit irrigation. Ren et al. (2008) also reported that the best simulated rainwater amount for the RF planting model is 230–440 mm, but the biomass accumulation of maize did not increase significantly when precipitation was higher than 440 mm. Many researchers are studying appropriate rainwater management through ridges covered plastic film mulching to improve WUE (Li et al., 2009). In addition to decreasing the ET rate, high WUE is endorsed to use deep soil moisture content to support winter wheat yields (Li et al., 2004; Duan et al., 2006).

### ET

It is well-known that the evaporation is inversely correlated to the depth of soil moisture contents (Tian et al., 2003). Under the RF planting model, the rainfall water infiltrated deeper and prolongs the period of moisture availability, meaning that there would be less evaporation. Plastic film mulching on ridges can decrease evaporation from the topsoil and can reduce total water utilization (Fan et al., 2002). Earlier studies confirmed that the RF planting model with simulated rainfall conditions significantly reduced the ET rate at the field scale and improved water use efficiency (Zhang et al., 2009). Various studies have shown that plastic film could significantly decrease evaporation rate, and thus increase WUE in semi-arid regions (Gan et al., 2013). Other studies have indicated, however, that as SWS increases under the RF system, more water would be consumed by crop transpiration, ultimately causing the ET rate to be higher (Qin et al., 2014).

### Grain filling process

Increasing the simulated rainfall level from 125 to 200 mm under different planting patterns and deficit irrigations, led to significant increases in maximum grain weights, and maximum and mean grain-filling rates for superior and inferior grains; however simulated rainfall increased from 200 to 275 mm did not significantly affect grain filling rates under different planting patterns (Table 2). Grain filling rates were significantly increased in the RF system compared to TF planting. At 200 mm precipitation, RF_150_ and TF_150_ treatments showed significant increases in maximum grain weights and maximum and mean seed-filling rates of superior and inferior grains, compared to RF_0_ and TF_0_ treatments; however, the RF system had no significant influences on these variables for the inferior grains. At the 275 mm simulated precipitation level, there were no significant differences between RF and TF planting models for these parameters under 150 and 75 mm deficit irrigation; however, significant variation was recorded between 0 and 75 mm or 150 mm irrigation under both planting patterns. Yang and Zhang (2010) also reported that winter wheat could not attain their potential yield due to the poor grain-filling rate of inferior grains. Earlier studies have recommended that the RF system with simulated rainfall had a significant influence on the grain-filling process of superior and inferior grains (Liu et al., 2013; Ali et al., 2016). Liu et al. (2015) determined that plastic film on ridges with deficit irrigation significantly improved the grain-filling rate of inferior grains of maize as compared to TF planting model; but, RF system did not have a significant influence on superior grains, which indicated that inferior grains are more subject to water stress. A previous study (Xu et al., 2007) showed that the SWS level may have specific effects on the filling of superior and inferior seeds. Ali et al. (2016) found that plastic mulching had a significant effect on seed filling in the superior, middle, and inferior seeds at various rainwater levels, probably due to the adequate SWS for maize seed filling under the 430 mm rainfall. Plastic film mulching significantly enhanced the soil water content and increased the filling rates of the inferior seeds. Thus, Xu et al. (2007) suggested that plastic film mulching combined with RF system could significantly enhance the seed-filling rate and seed weight for inferior seeds in rice compared with conventional planting, but this method had no significant effects on the superior rice seeds, thereby demonstrating that inferior seeds are more susceptible to water scarcity than superior seeds.

### Winter wheat yields WUE, RIWUE, and RIWP

The interaction between planting patterns and deficit irrigation are the key features that effect winter wheat yields (Albrizio et al., 2010). The RF system can make better use of precipitation, whereas deficit irrigation can also supply water at main growth stages as a result of improved total dry matter and grain yield (Li et al., 2009). Plastic film mulching with limited irrigation reduces soil evaporation, decreases the ET rate, and thus changes water utilization from soil water evaporation to crop transpiration, increasing wheat yields (Fang et al., 2006). Winter wheat grown under the RF planting model with extra water input, such as deficit irrigation, will reached to potential yield, which will decrease the cost to profit ratio of extra water input (Zhang et al., 2007). In our study, we found that the RF system improved SWS and decreased evaporation, and the grain yield was 0.82 t ha^−1^ (19%) higher than that of TF planting. The average mean over 2 years indicated that grain yield with RF1_150_; RF1_75_ and RF1_0_ were significantly improved by 0.73, 0.77, and 0.88 t ha^−1^, respectively, compared to TF1_150_, TF1_75_, and TF1_0_, respectively. Compared with TF2_150_, TF2_75_, and TF2_0_ treatments mean grain yield of RF2_150_, RF2_75_, and RF2_0_ treatments was significantly increased by 1.19, 1.15, and 1.13 t ha^−1^, respectively. Average grain yield was significantly increased in RF3_150_, RF3_75_, and RF3_0_treatments by 0.59, 0.29, and 0.77 t ha^−1^, respectively, compared with TF3_150_, TF3_75_, and TF3_0_ treatments, respectively. Ding et al. (2007) also revealed that the RF planting model improved the seed yield by 83.8–178.9% compared with TF planting model. Li and Gong (2002) found no need to implement the RF system in areas with >400 mm of precipitation because the water requirement of millet is only 300 mm. Ren et al. (2008) also recommended that the increased yield in maize under the RF system is decreased when the simulated precipitation beyond 400 mm.

Enhancing WUE is the main goal for researchers (Deng et al., 2006), and maximum WUE under the RF system is attained mostly by reducing the ET rate at the field scale (Albrizio et al., 2010). In our study, the traditional flat planting pattern under different deficit irrigations with simulated rainfall conditions led to a higher ET rate than the RF system. The average mean over 2 years showed that the RF system increased soil moisture content and reduced evaporation; the ET rate was 109 mm (41%) lower than that of TF planting pattern. The mean of ET over two years indicated that the ET rates of RF1_150_, RF1_75_, and RF1_0_ were significantly (*P* < 0.05) reduced by 128 mm (28%), 123 mm (28%), and 95 mm (24%), respectively, compared to TF1_150_, TF1_75_, and TF1_0_, respectively. Compared with TF2_150_, TF2_75_, and TF2_0_ treatments mean ET over 2 years for RF2_150_, RF2_75_, and RF2_0_ treatments were significantly reduced by 127 mm (33%), 130 mm (34%), and 96 mm (29%), respectively. The optimum rainfall amount for the RF planting model is 230–440 mm and there are no significant increases in WUE when the precipitation goes beyond 440 mm (Li X. Y. et al., 2001). In our study, WUE and grain yield had a tendency to improve with the simulated rainfall and deficit irrigations levels, but there were no significant differences when the simulated rainfall was higher than 200 mm under both planting patterns. As grain yield increased, WUE increased. WUE increased by 8.10 kg mm^−1^ ha^−1^ (70.67%) under the RF system compared to TF planting, due to a decreased ET rate at the field scale. Average WUE of RF2_150_, RF2_75_, and RF2_0_ was significantly improved by 12.39, 11.29, and 8.00 kg mm^−1^ ha^−1^, respectively, compared to TF2_150_, TF2_75_, and TF2_0_, respectively.

The average mean over two years data shows that the RF system significantly improved RIWUE 3.44 kg mm^−1^ (22.50%) compared to TF planting pattern (Figure 4). In addition, the RF2_150_ treatment achieved a maximum grain yield by using less irrigation and rainfall water. As a result, the RIWUE in the RF2_150_ treatment was 3.35 and 5.09 kg mm^−1^ higher than RF1_150_ and TF1_150_ treatments, respectively (Figure 5). Wu et al. (2015) revealed that the water use was reduced but the yield was increased with the RF system treatments, thus the WUE increased significantly by 15.77 and 19.82%, and the IWUE improved by 2.1 and 2.2 times, compared with FI (furrow irrigation) and BI (border irrigation), respectively. The RIWP shows the capacity of winter wheat seed yield to increase due to the addition of simulated precipitation and irrigation water, and the RF2_150_ treatment in 2015–16 and RF2_150_ and TF2_150_ treatments in 2016–17 had a satisfactory, efficient use of simulated rainfall, and deficit irrigation was reduced by half (Figure 5). Wu et al. (2015) also revealed that RF system enhanced IWP by 1.71 and 5.70 times compared with FI and BI in 2011. But RF system significantly decreased IWP with IAF (irrigation during flowering stage) in 2012, it can be explained that the frequent rainfall in August 2012 intensified the superiority of water harvesting of RF system, in consequence, the irrigation effects was weaken. Thus, the application of RF system is closely related to the rainfall condition and irrigation scheduling.

### Economic returns

Financial profit is a valuable indicator when evaluating water saving agricultural water management strategies (Li et al., 2016). The RF planting model requires a higher investment at the outset, but the system increases both crop yields and net income (Ren et al., 2008). Zhang et al. (2017) also revealed that RF system significantly improved both crop yields and economic benefits 2,700 Chinese Yuan ha^−1^ than that of TF planting. We found that the RF system with simulated precipitation amounts under deficit irrigation had positive effects on net income; however, excessive simulated rainfall amounts and deficit irrigation led to be a misuse of water resources in semi-arid regions leading to lower net income (Table 5). The RF2_150_ treatment had a significantly positive effect and led to the highest net income (12,593 CNY ha^−1^), output/ input ratio (2.65), and net income difference (10,052 CNY ha^−1^) among all treatments. Thus, we recommend the RF2_150_ treatment as a well-organized practice for increasing wheat productivity in the dry-land farming system of China.

## Conclusion

The semi-arid areas of China are faced with serious water shortages, and irrigation for maintaining agricultural productivity is becoming costly and inadequate. Thus, improving water-saving farming systems is crucial to resolve the water scarcity problem that affects crop productivity in semi-arid regions. In the current study, we investigated the RF system with three simulated rainfall amounts and three levels of deficit irrigation. We found that the RF system with 200 mm simulated precipitation and 150 mm deficit irrigation had a significant effect on SWS in the depth of (0–200 cm), reduced ET at the field scale, improved total biomass per plant, grain filling rate, and led to water saving benefits and higher grain yields, consequently achieving a higher WUE and RIWP. We found that simulated rainfall and deficit irrigation have significant effects on dry matter accumulation, yields, and RIWP. The grain yield increased significantly under RF2_150_ treatment, but there were no significant increases in grain-filling rates, grain weight of superior and inferior grains, or net economic profit as simulated rainfall increased from 200 to 275 mm. Based on our consideration of WUE, RIWP, total dry matter accumulation, and net economic profits, we recommend that the RF2_150_ treatment as an well-organized practice for increasing wheat productivity in the dry-land farming system of China.

## Author contributions

The manuscript was reviewed and approved for publication by all authors. ZJ, XR, and TC conceived and designed the experiments. SA, YX, and XM performed the experiments. SA, IA, and QJ analyzed the data. SA and YX wrote the paper. ZD, PZ, TC, MK, IA, XR, and ZJ reviewed and revised the paper. SA and ZJ corrected the English language for the paper.

### Conflict of interest statement

The authors declare that the research was conducted in the absence of any commercial or financial relationships that could be construed as a potential conflict of interest.
